# Descemet membrane endothelial keratoplasty in cases with existing scleral-sutured and iris-sutured intraocular lenses

**DOI:** 10.1186/1471-2415-14-6

**Published:** 2014-01-20

**Authors:** Daniel Röck, Tobias Röck, Karl-Ulrich Bartz-Schmidt, Efdal Yoeruek

**Affiliations:** 1 Department of Ophthalmology, Eberhard-Karls University, Schleichstr 12, Tüebingen 72076, Germany

**Keywords:** Descemet membrane endothelial keratoplasty (DMEK), Aphakia, Bullous keratopathy, Transscleral-fixated intraocular lens (sf-IOL), Iris-fixated intraocular lens (if-IOL)

## Abstract

**Background:**

To report two cases of Descemet Membrane Endothelial Keratoplasty (DMEK) in patients with existing scleral-fixated and iris-fixated intraocular lenses (sf-IOL and if-IOL, respectively).

**Case presentation:**

DMEK procedures were performed on a 49-year-old woman with a pre-existing sf-IOL (case 1) and a 69-year-old woman with a pre-existing if-IOL (case 2) in order to treat secondary corneal edema due to pseudophakic bullous keratopathy. Visual acuity, refractive error, intraocular pressure, slit lamp examination, pachymetry measurements and endothelial cell density (ECD) were considered and repeated during follow-ups.

Both cases had no intraoperative complications. At postoperative day 1 graft centration and complete attachment were noted. The IOL positions were unchanged in comparison to their preoperative positions. In case 1, visual acuity improved from 1/15 at 1 meter preoperative to 20/200 within one week and to 20/63 within 12 weeks of follow up. In case 2, visual acuity improved from counting fingers at 1 meter preoperative to 20/200 within one week and to 20/100 within 12 weeks of follow-up. In case 2 a partial graft dislocation was observed at postoperative day twenty. Complete graft re-apposition was achieved by rebubbling procedure performed with intracameral air injection.

**Conclusions:**

DMEK surgery in the treatment of pseudophakic bullous keratopathy in the presence of sf-IOL and if-IOL can successfully be performed. These eyes are at increased risk of IOL dislocation into the vitreous cavity during DMEK surgery.

## Background

Recently novel techniques of posterior lamellar keratoplasty for the treatment of corneal endothelial diseases (CED) have been developed [1-3]. Lamellar keratoplasty has been shown to offer a promising alternative to penetrating keratoplasty (PK) and has become a popular procedure for the management of CED [2-5].

Descemet Membrane Endothelial Keratoplasty (DMEK) offers several advantages over PK such as its minimal invasiveness, rapid visual rehabilitation, excellent uncorrected and corrected visual acuities and reduction of postsurgical atigmatism [6-8].

Despite the obvious superiority over PK in CED, adequate visualization of the anterior chamber is an important prerequisite to a successful DMEK. The donor tissue’s properties make it liable to intraoperative loss. This is especially true in eyes with extensive iris defects and dilated pupils. The relatively thin donor tissue increases the intraoperative difficulty. The donor tissue is so thin that it can be difficult to unfold and orientate properly onto the donor bed.

Moreover, DMEK may be relatively contraindicated in eyes with unpressurizable anterior chambers [9]. Thus, it may be challenging to perform a DMEK on postvitrectomy eyes and ones with potentially unstable IOLs, such as scleral-fixated and iris-fixated lenses (sf-IOL and if-IOL, respectively). Filling the anterior chamber with air at the end of the procedure could potentially dislocate an unstable IOL.

We present two cases of successful DMEK surgery performed in eyes with pre-existing scleral-sutured and iris-sutured IOLs, that therefore present an increasd risk of IOL dislocation.

## Methods

### Donor preparation

The donor corneoscleral rim was placed on a sterile circular surface and was scored and stained with trypan blue to highlight the scoring mark; thereafter, it was placed in a corneal viewing chamber containing corneal storage solution (Culture Medium I; Biochrom AG, Berlin, Germany). A circular incision with a hockey knife was made. Complete dissection of the DMEK tissue from the corneoscleral rim was achieved by grasping the peripheral free tissue flap using untoothed curviliniear forceps specially developed for this task by Yoeruek [10]. A spatula was used to lift the trephinated graft off the stromal bed after complete dissection and trephination. The DM was placed in culture medium before the surgery. At surgery the culture medium was carefully drained and the DMEK roll was thoroughly rinsed with BSS. To open the tissue and create a double roll a direct flow on the top of the tissue with BSS was applied. The tissue was then stained with trypan blue.

The age of the donor in case one was 50 years and in case two 57 years. The mean cellular endothelial count of the tissue was 2700 cells per square millimeter and 2400 cells per square millimeter in case one and two.

### Recipient preparation, graft insertion, and positioning

The surgical technique involved the initial placement of two paracentheses in the 2-o’clock and 10 o’clock positions. To remove the recipients’ DM, proper visualization of the anterior chamber using air pressurized at 30 mmHg is needed. This is followed by the introduction of a reversed Sinskey hook through a paracenthesis for Descemetorhexis. A 2.75 mm clear corneal tunnel was created with a 2.75 mm slit knife at the 12 o’clock position. The dissected donor DM was loaded into a shooter (DMEK shooter Geuder AG, Heidelberg, Germany) in the double-roll-form. The injector was turned so that the double roll was facing upward. Implantation took place into a soft eye. After confirmation of orientation, primary using Melles rule of the rolled edges with the endothelium facing outward the anterior chamber was obliterated completely via the paracentheses. No air was injected above or below the DM (to aid in the process of unfolding). The eye was kept in the soft state, and digital pressure was applied at the equatorial plane, thereby preventing any refolding or recurling. Apposition and centration was achieved because of the shallow anterior chamber, the soft eye status, and the corneal tapping in combination with equatorial digital pressurization. After complete unfolding, the infusion of air was placed in the anterior chamber from an infusion pump connected to a 30-gauge cannula. Air is infused into the anterior chamber below the DM at a continuously regulated pressure of 30 mmHg for final DM fixation [11].

## Case presentation

### Patient 1

**Figure 1:**
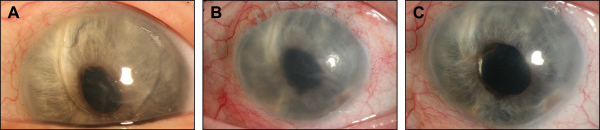
**DMEK surgery with pre-existing scleral sutured IOL. A**, Preoperative status demonstrating secondary corneal edema due to bullous keratopathy because of an anterior chamber IOL. **B**, Diffuse corneal edema and a stable, well-positioned, scleral-fixated IOL after explantation of the anterior chamber IOL. **C**, Appearance 3 months after DMEK surgery, showing a clear cornea with a well-centered and well-attached graft and unchanged position of the IOL.

In 2011 a 49-year-old woman was referred with a complaint of worsening blurry vision and discomfort in the left eye because of corneal decompensation after anterior chamber IOL implantation with secondary glaucoma. Anterior segment examination showed diffuse corneal edema, descemet membrane folds and an anterior chamber IOL (Figure 1A). 20 years earlier, she had a bilateral cataract extraction with an anterior chamber IOL implantation. The left eye had a corrected visual acuity of 20/200. A clinical diagnosis of corneal decompensation with bullous keratopathy was made, and IOL explantation was discussed and agreed. Three months after explantation of the anterior chamber IOL and a limited anterior vitrectomy a scleral-fixated IOL was implanted. The IOL was fixated using a knotless zigzag-shaped intrascleral suture (Z-suture) [12]. After this surgery best-corrected visual acuity (BCVA) in her left eye was 1/15 at one meter with 0 sph = -3.00 cyl 131°. Anterior segment examination showed diffuse corneal edema and a stable, well-positioned, scleral-fixated IOL (Figure 1B). A DMEK procedure was performed. The postoperative course was normal. BCVA had improved by 3 months to 20/63 without any changes in refraction. The cornea was clear with a well-centered and well-attached graft, and the scleral-fixated IOL was in place and well-centred (Figure 1C). The baseline donor endothelial cell densitiy decreased from 2700 cells per square millimeter preoperative to 1900 cells per square millimeter 3 months after surgery. The limiting factor of VA was a pre-existing advanced glaucoma.

### Patient 2

**Figure 2:**
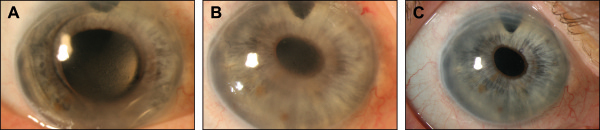
**DMEK surgery with pre-existing iris-sutured IOL. A**, Preoperative status demonstrating secondary corneal edema due to bullous keratopathy because of an anterior chamber IOL and showing surgical peripheral iridectomy at 12 o’clock. **B**, Diffuse corneal edema and a stable, well-positioned, Artisan iris-fixated IOL after explantation of the anterior chamber IOL. **C**, Appearance 3 months after DMEK surgery, showing a clear cornea with a well-centered and well-attached graft and unchanged position of the IOL.

A 69-year-old woman suffering from bullous keratopathy after cataract surgery with implantation of an anterior chamber IOL in her right eye was referred to our institution. In November 2010 at the time of presentation anterior segement examination showed diffuse corneal edema, a well-positioned anterior chamber IOL and a surgical peripheral iridectomy at 12 o’clock (Figure 2A). Prevention of further endothelial cell loss and eventual graft failure would require removal of the anterior chamber IOL and exchange for a posterior chamber IOL. The non-foldable IOL was removed a limited anterior vitrectomy was made and the anterior chamber IOL was exchanged with an Artisan iris-fixated posterior chamber IOL. At this time the BCVA in her right eye was 20/160. During a follow-up of a year, bullous keratopathy developed, causing a decrease in BCVA from 20/160 to counting fingers at 1 meter (Figure 2B). A DMEK procedure was performed by using the technique previously described in the right eye. On the first postoperative day, the graft was well positioned and nearly complete attached, with anterior chamber air-fill of nearly 30%. Three weeks after DMEK a rebubbling procedure was performed with intracameral air injection because of a partially detached graft. BCVA had improved by 3 months to 20/100 (Figure 2C). The baseline donor endothelial cell densitiy decreased from 2400 cells per square millimeter preoperative to 1600 cells per square millimeter 3 months after surgery. The limiting factor of VA was an age-related macular degeneration.

## Conclusions

Today, several techniques and different IOL types to treat aphakia have been described. The iris may serve as one point of fixation exemplified by an if-IOL (e.g. Artisan lens). Two options are available, either the anterior or the posterior approach. Because the Artisan lens is not foltable, the use of this lens is restricted to eyes undergoing extended corneoscleral incision with a standard 5.5- to 6-mm corneoscleral tunnel incision at the 12-o’clock position.

In cases of inadequate capsular support, sulcus lenses may be fixated to the sclera. Since the early 1990s, the ab-externo technique of Lewis and the refined ab-interno technique of Smiddy et al are the basis of multiple variations in transscleral suturing [13,14]. Szurman et al. modified this technique into a knotless one without direct knot exposure and, hence, lessened complications [12].

The concept of DMEK surgery has been proven by several studies. During the surgery two critical steps, first descemetorhexis under air visualization and second final fixation under air pressurization, pose an increased risk of IOL dislocation especially in cases having if-IOL and sf-IOL implants. When these two steps are preformed there is a risk that the air bubble could exert pressure on the IOL, endangering its stability, with possible malpositioning or even dislocation into the vitreous chamber. The surgeon has to consider these eventualities wisely. Additionally, in post-vitrectomy and sf-IOL cases the air passage is relatively unhindered into the posterior chamber. Air migration into this space may result in extensive irido-corneal adhesions and possibly severe ocular hypertension. Furthermore, considerable mechanical trauma may also be caused at the posterior aspect involving the delicate point of fixation [15]. Air pressure exerted onto the IOL’s surface endangers its stability with subluxation or even dislocation affecting the lenticular system which is especially vulnerable in the early postoperative period.

Lapenna et al. reported a case with dis-enclavation of an iris claw IOL after Descemet Stripping Automated Endothelial Keratoplasty (DSAEK) [15]. Tay et al. have described a similar case in which a silicone plate haptic posterior chamber IOL dislocated into the vitreous cavity after DSAEK in the presence of a posterior capsulotomy [16].

However, a direct comparison of DSAEK and DMEK in this respect would be incorrect, as the graft’s properties result in longer OR time when using DMEK, especially when applied to vitrectomized eyes. The presence of a thin stromal layer in DSAEK allows for ease of handling of the donor tissue. In absence of the stromal layer the tendency of the donor tissue to roll onto itself makes proper positioning and fixation difficult.

In order to address this issue conveniently, a special maneuver was introduced by us. The maneuver offers complete unfolding of the DM without the need of an additional air bubble above or below the graft. Air is only introduced for the apposition and final fixation. Unfolding was achieved with simultaneous digital pressure in the equatorial region and tapping of the corneal surface. It is an especially helpful maneuver in eyes with a deep anterior chamber as in postvitrectomy eyes [11].

In conclusion, the presented cases demonstrate the feasibility of DMEK in eyes with sf-IOLs and if-IOLs. The successful outcomes imply that DMEK is not “absolutely contraindicated” in such cases. Nevertheless, a high level of caution is needed in both critical steps as mentioned above. Further studies with a larger series and longer follow-up are required to quantify the complication rates of DMEK in eyes with sf-IOLs and if-IOLs.

## Consent

Written informed consent was obtained from the patient for publication of this case report and any accompanying images. A copy of the written consent is available for review by the Editor-in-Chief of this journal.

## Competing interests

The authors declare that they have no competing interests.

## Authors’ contributions

DR participated in management of the cases, analyzed the data, and drafted the manuscript. TR was involved in drafting this manuscript. EY was the attending surgeon for the cases and revised the manuscript. KUBS has given final approval. All authors read and approved the final manuscript.

## Pre-publication history

The pre-publication history for this paper can be accessed here:

http://www.biomedcentral.com/1471-2415/14/6/prepub
